# Immunoexpression of Interleukin-22 and Interleukin-23 in Oral and Cutaneous Lichen Planus Lesions: A Preliminary Study

**DOI:** 10.1155/2013/801974

**Published:** 2013-11-26

**Authors:** Jun Chen, Jinqiu Feng, Xiangdong Chen, Hui Xu, Zengtong Zhou, Xuemin Shen, Zhexuan Bao, Wei Liu, Zhengyu Shen

**Affiliations:** ^1^Department of Dermatology, Ninth People's Hospital, Shanghai Jiao Tong University School of Medicine, 639 Zhizaoju Road, Shanghai 200011, China; ^2^Department of Preventive Dentistry, Shanghai Stomatological Disease Center, Shanghai 200001, China; ^3^Shanghai Key Laboratory of Stomatology, Ninth People's Hospital, Shanghai Jiao Tong University School of Medicine, 639 Zhizaoju Road, Shanghai 200011, China

## Abstract

Interleukin- (IL-) 22 is the signature cytokine of T-helper (Th) 22 cells, and IL-23 is required for IL-22 production. The objective of this study was to examine the immunoexpression of IL-22 and IL-23 in archival paraffin-embedded biopsy specimens from oral LP (*n* = 42) and cutaneous LP (*n* = 38) against normal control tissues. The results showed that the percentage of cells expressing IL-22 and IL-23 in LP were significantly higher in LP compared to controls, respectively (both *P* < 0.001). The correlation between IL-22 and IL-23 expression was significant (*P* < 0.05). Moreover, the percentage of cells expressing IL-22 and IL-23 in oral LP were significantly higher than cutaneous LP (*P* < 0.05). Collectively, our findings demonstrated that the increased expression of IL-22 and IL-23 in LP lesions could play roles in the pathogenesis of LP. Moreover, oral LP expressing IL-22 and IL-23 was higher than cutaneous LP, probably due to Th22 cells as an important component of oral mucosal host defense against oral microbiota and tissue antigens. This may be associated with the difference in clinical behaviour of the two variants of the disease.

## 1. Introduction

Lichen planus (LP) that is a relatively common chronic inflammatory mucocutaneous disease of probable immune-based aetiology, involves the oral and genital mucosal surfaces, skin, nails, and scalp. LP is characterized by a T-cell-mediated immune response against epithelial cells, causing epithelial cell damage and subepithelial band-like infiltration of T lymphocytes. The mechanisms involved in this disease remain unclear [1–3]. Although oral and cutaneous LP share similar histologic features, they are distinguished by heterogeneity of the clinical behaviour. Oral LP follows a chronic and recalcitrant course and may persist for very long periods, with alternating periods of exacerbation and quiescence, and those atrophic, erosive, or bullous areas are often painful and sensitive, while cutaneous LP tends to be self-limited regardless of therapy [4–6]. Local differences in the immune-related molecules could help to explain the observed variation in clinical behavior of oral mucosa and skin lesions, whereas limited data are available so far on these molecules in the previous studies [7–10].

Interleukin- (IL-22) is the signature cytokine of T-helper (Th) 22 cells, which are considered to be a newly found CD4+ Th subset [11]. IL-22 has recently been involved in the pathogenesis of autoimmune and inflammatory disorders such as psoriasis, lupus erythematosus, and rheumatoid arthritis [12]. In addition, Th22 cells are important contributors to mucosal host defense, and IL-22 is central to host protection against bacterial infections at barrier sites [13]. Recent studies revealed that IL-23 is required for IL-22 production, and IL-23 is also regarded as a pivotal cytokine for the pathogenesis of inflammatory and autoimmune diseases [14, 15]. In addition, mice deficient for IL-23 fail to resist infection by intestinal or pulmonary bacterial pathogens [16]. IL-22 is a downstream effector cytokine of IL-23 [17]. Whether IL-22 and IL-23 may be implicated in the local immune response observed in the tissue samples of patients with LP is, however, still unknown.

We thus hypothesized that IL-22 and IL-23 expression in oral and cutaneous LP lesions would be dysregulated and distinct, reflecting potential differences in their immunopathogenesis, and that IL-23/IL-22+ Th22 cells may also play roles in the development and maintenance of LP. In this study, we examined the immunoexpression of IL-22 and IL-23 in archival formalin-fixed paraffin-embedded (FFPE) biopsy specimens from 80 cases of LP (oral LP, *n* = 42; cutaneous LP, *n* = 38), comparing the results with those of normal control tissues (oral mucosa, *n* = 10; skin, *n* = 10), and evaluated whether both proteins are significantly involved in the difference in immunopathologic behaviour of the two variants of the disease.

## 2. Materials and Methods

### 2.1. Subjects and Tissue Specimens

Three pilot case-control study designs were used in the current study. (i) The first setting included 42 patients with oral LP and 10 gender- and age-matched healthy individuals undergoing orthognathic surgery as control. (ii) The second setting included 38 patients with cutaneous LP and 10 gender- and age-matched healthy individuals undergoing plastic surgery as control. (iii) The third setting included these 42 patients with oral LP versus 38 gender- and age-matched patients with cutaneous LP. The characteristics of study subjects are presented in Table 1. FFPE tissue specimens of 5 *μ*m thick were prepared from the biopsies and subjected to routine hematoxylin and eosin staining to histopathologic examination.

The enrolled patients with oral and cutaneous LP were diagnosed clinically and confirmed histopathologically, and all lesions were characterized by similar degree of inflammatory activity according to the criteria recommended in the published literature [7, 18, 19]. The patients were recruited based on the inclusion and exclusion criteria described previously [8]. Patients with diabetes, hypertension, infectious, and allergic disorders or other autoimmune diseases, such as psoriasis, rheumatoid arthritis, and lupus erythematosus, were excluded. Furthermore, these patients had not received treatment for LP, and healthy individuals had no disorders known to affect their immune function. This study was approved by our local Ethics Committee (number 201202) with informed consent obtained from all participating subjects.

### 2.2. Immunohistochemical Analysis

Tissue sections (5 *μ*m thick) from FFPE blocks of those samples were mounted on positively charged glass slides. Immunohistochemical staining was done by using the Leica Automatic Stainer (Leica Microsystems, Wetzlar, Germany) and the manufacturer's protocol. The goat IL-22 antibody (dilution 15 *μ*g/mL; catalogue number AF782; R&D Systems Inc.) was used to detect IL-22 expression, and the mouse IL-23p19 antibody (dilution 8 *μ*g/mL; catalogue number HLT2736; BioLegend Inc.) was used to detect IL-23 expression. We performed immunohistochemistry with negative and positive controls for IL-22 and IL-23, respectively. In negative controls, the primary antibody was replaced by nonimmune IgG of the same isotype to ensure specificity. The inflamed salivary glands of patients with Sjögren's syndrome with known immunopositivity for IL-22 [17] and IL-23 [20] protein as previously reported were used as positive controls in each batch of sections analyzed.

IL-22 and IL-23 expression were evaluated separately in the epithelial and subepithelial compartments of LP. Cell cytoplasmic and/or membrane immunoreactivity was considered to indicate positive expression. The quantitative evaluation of immunoreactivity was performed according to criteria of staining intensities described by Santoro et al. [9, 10]. Briefly, 5 digital images of high-power fields at 400X magnification selected randomly on immunostained sections were captured by the DP 70 CCD camera (Olympus, Tokyo, Japan). Then, positive cells were counted using Image Pro-Plus software (version 6.0, Media Cybernetics, CA, USA) in this study.

### 2.3. Statistical Analysis

Statistical analysis was performed by using the unpaired Student's *t*-test for quantitative variables of IL-22 and IL-23 positive expression in the epithelial and subepithelial compartments. Spearman correlation coefficient was used to determine the correlation between IL-22 and IL-23 expression for all cases. Two-sided *P* values were calculated, and *P* value of <0.05 was accepted for statistical significance.

## 3. Results

### 3.1. IL-22 and IL-23 Expression in LP Lesions and Normal Control

**Figure 1:**
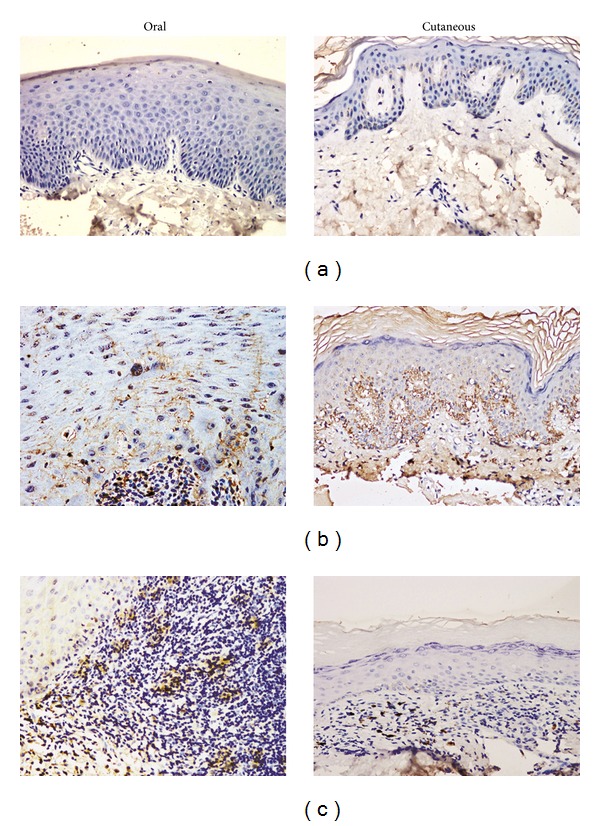
Representative expression pattern of IL-22: (a) negativity in normal oral mucosa and normal skin; (b) positivity in epithelial cells of oral and cutaneous lichen planus (LP); (c) positivity in subepithelial cells of oral and cutaneous LP. Magnification, ×200.

**Figure 2:**
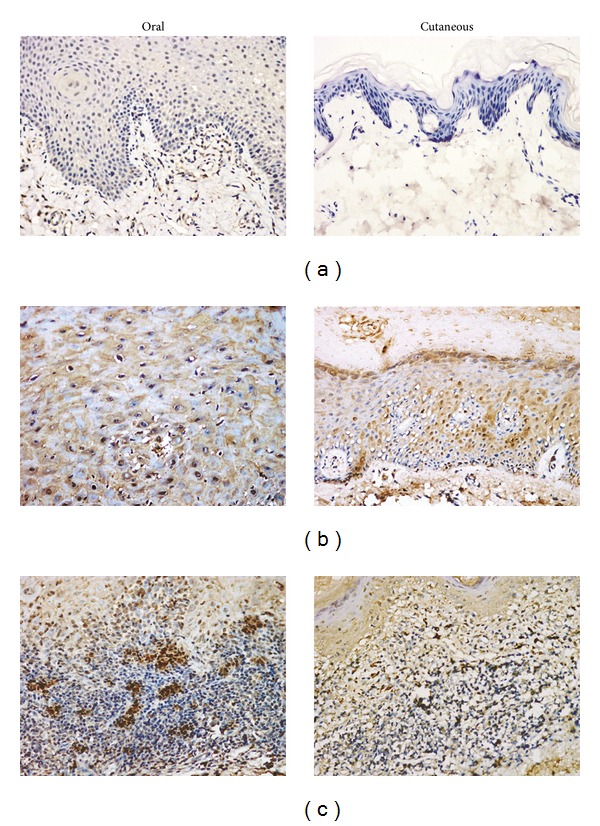
Representative expression pattern of IL-23: (a) negativity in normal oral mucosa and skin; (b) positivity in epithelial cells of oral and cutaneous lichen planus (LP); (c) positivity in subepithelial cells of oral and cutaneous LP. Magnification, ×200.

Representative positive immunoexpresion of IL-22 and IL-23 in specimens of epithelial cells and subepithelial lymphocyte infiltration of LP and normal controls was shown in Figures 1 and 2. The quantification expression levels of IL-22 and IL-23 in specimens were presented in Table 2.

In normal control, cells expressing each molecule were rare or absent; the few positive cells were located in the epithelial and subepithelial layers. In the epithelial and subepithelial compartments of LP, the percentage of cells expressing IL-22 in general LP (*n* = 80) were significantly higher compared to normal controls (*n* = 20), respectively (both *P* < 0.001). In the epithelial and subepithelial compartments of LP, the percentage of cells expressing IL-23 in general LP (*n* = 80) were also significantly higher compared to normal controls (*n* = 20), respectively (both *P* < 0.001). Moreover, a positive correlation between IL-22 and IL-23 expression in the epithelial (*P* = 0.025; correlation coefficient, 0.251) and subepithelial layer (*P* = 0.001; correlation coefficient, 0.370) was found to be significant, respectively.

Separately, in the oral LP (*n* = 42), IL-22 expression in the epithelial (*P* = 0.024) and subepithelial (*P* < 0.001) layers was significantly higher compared to normal oral mucosa (*n* = 10). Meanwhile, IL-23 expression in the epithelial (*P* < 0.001) and subepithelial (*P* < 0.001) layers was also significantly higher compared to normal oral mucosa (*n* = 10). As for cutaneous LP (*n* = 38), IL-22 expression in the epithelial (*P* < 0.001) and subepithelial (*P* < 0.001) layers was significantly higher compared to normal oral mucosa (*n* = 10). Meanwhile, IL-23 expression in the epithelial (*P* < 0.001) and subepithelial (*P* < 0.001) layers was also significantly higher compared to normal oral mucosa (*n* = 10).

### 3.2. IL-22 and IL-23 Expression in Oral versus Cutaneous LP Lesions

To investigate whether immune molecules expression in oral and cutaneous LP was distinct, we compared IL-22 and IL-23 expression in the epithelial and subepithelial compartments. Although IL-22 expression in the epithelial layer was not significant (*P* > 0.05), its expression in the subepithelial layer of oral LP was significantly higher (2.6 times more) than cutaneous LP (*P* = 0.036). Meanwhile, IL-23 expression in the epithelial layer of oral LP was significantly higher (3.6 times more) than cutaneous LP (*P* = 0.003), and its expression in the subepithelial layer of oral LP was significantly higher (2.6 times more) than cutaneous LP (*P* = 0.006).

## 4. Discussion

The identification of Th22 cells as a newly discovered distinct subset of CD4+ T cells has extended the Th1/Th2 paradigm in the adaptive immunity and plays a role in the development of autoimmune and inflammatory disorders. IL-22 is the signature cytokine of Th22 cells, and IL-23 is required for IL-22 production [11–14]. LP is characterized by a T-cell-mediated immune response against epithelial cells, causing epithelial cell damage and subepithelial infiltration of T lymphocytes. To our knowledge, there has been only one English literature on serum IL-23 in oral LP patients studied by Wang et al. [21]. They reported an increased expression level of serum IL-23 by using ELISA in patients with oral LP concomitant chronic periodontitis compared with healthy controls [21]. Hardly any reports that are available so far on IL-22 and IL-23 may be implicated in the local immune response observed in the tissue samples of patients with LP.

In the current study, we presented new information that (i) IL-22 expression level in the oral and cutaneous LP lesions was increased compared to normal controls; (ii) IL-23 expression level in the oral and cutaneous LP lesions was increased compared to normal controls; (iii) the significant correlation between IL-22 and IL-23 expression in LP lesions infiltration was found. These results suggested that the two major immune molecules may play roles in the pathogenesis of LP, enhancing our understanding of the inflammatory response in LP.

In this study, we further focused on the differences of the clinical behaviour of oral and cutaneous LP. A majority of inflammatory diseases of the oral mucosa and skin are due to T-cell-mediated immune response. Comparative studies of the mechanisms by which mediators react in these tissues should be performed in this context. LP affecting the oral mucosa and skin manifests distinct clinical appearances in the diferent organs, and this is probably because of variations in their structure and function. Oral mucosa and skin differ in keratinization patterns, resistance to external pressure, and moist versus dry environment. Moreover, oral mucosa is exposed to large amounts of antigens, whether from food, bacteria, virus, or fungi, compared to the skin. It is possible that this antigenic load interferes with its immunocompetent cells.

To further explore if the immunocompetent molecules could account for heterogeneity of clinical behavior, we evaluated IL-22 and IL-23 expression in a series of oral versus cutaneous LP. Noteworthy, we found another new information that the expression levels of IL-22 and IL-23 significantly increased in oral LP compared with cutaneous LP. This indicates that IL-23+/IL-22+ Th22 cells were increased significantly in oral LP compared with cutaneous counterpart. Van Belle et al. [13] recently established Th22 cells as an important component of mucosal antimicrobial host defense. IL-22 is central to host protection against bacterial infections at barrier sites [16], and IL-22 production was strictly IL-23 dependent [14]. As it was explained above, the increased levels of IL-22 and IL-23 in oral LP might be due to action of the oral microbiota and tissue antigens of uncertain origin that initiate and maintain the inflammatory response.

Taken together, these data offer a possible explanation for the difference of IL-23/IL-22 pathway in clinical behaviour of the two variants of the disease and support the hypothesis that different immunopathogenetic mechanisms might be involved in the two variants of OLP. In addition, it should be noted that these results are not influenced by therapy, since no patients received treatments known to influence immune system (such as steroids) before biopsy. As proposed for other chronic autoimmune disorders associated with IL-23/Th22 cells [22, 23], the involvement of IL-23/IL-22 pathway in the pathogenesis of LP could be considered for selective therapeutic inhibitory targeting.

LP is characterized by a T-cell-mediated immune response, and both IL-22 and IL-23 are important mediating cytokines for these responses. IL-22 is a downstream effector cytokine of IL-23 [17]. The role of IL-23/IL-22 in pathogenesis of LP lesion is still unknown. Based on the results of the current study, we briefly hypothesise that IL-22 can be expressed by Th22 cells in an IL-23-dependent fashion. Secreted by a newly found CD4+ T-helper subset called Th22 subset, IL-22 was found to mediate infiltration of T lymphocytes and epithelial cell damage by binding to the IL-22 receptor in epithelial cells. Moreover, IL-22 mediates early host defense against attaching and effacing bacterial pathogens [16]. IL-22 may also be involved in defense against oral microbiota and tissue antigens in oral LP.

We are aware of the limitations of our study as only immunohistochemistry is used to measure IL-22 and IL-23 expression, partly because only archival paraffin-embedded biopsy clinical specimens were available. Although oral LP is common, cutaneous LP is not a common lesion. New biopsies and PBMC of patients are collected for performing qPCR, ELISA, and FACS for IL-22 and IL-23 and investigating the immunopathologic mechanisms and therapeutic target of IL-23/IL-22 pathway in LP in the further studies.

In summary, our findings demonstrated that the increased expression of IL-22 and IL-23 in LP lesions including oral and cutaneous variants and their patterns of expression positively correlated with each other. Moreover, oral LP expressing IL-22 and IL-23 was higher than cutaneous LP, probably due to Th22 cells as an important component of oral mucosal host defense against oral microbiota and tissue antigens. This may be associated with the difference in clinical behaviour of the two variants of the disease.

## Figures and Tables

**Table 1:** Baseline characteristics of the study patients with lichen planus (LP) and controls.

Diagnosis	Number of cases	Mean ± SD age (years)	Age range (years)	Number of females	Number of males
Normal oral mucosa	10	45.2 ± 12.1	23~68	5	5
Oral LP	42	50.1 ± 10.6	22~74	21	21
Normal skin	10	49.6 ± 13.7	26~66	5	5
Cutaneous LP	38	50.0 ± 15.9	19~72	17	21

**Table 2:** Expression of IL-22 and IL-23 in normal oral mucosa and skin and lichen planus (LP) samples*.

	Normal control	General LP		Normal mucosa	Oral LP (OLP)		Normal skin	Cutaneous LP (CLP)		OLP versus CLP
Variable	*n* = 20	*n* = 80	*P* value	*n* = 10	*n* = 42	*P* value	*n* = 10	*n* = 38	*P* value	*P* value
IL-22										
Epithelial	3.58 (1.22)	107.04 (20.43)	<0.001	5.67 (2.19)	145.45 (36.38)	0.024	1.70 (0.98)	64.58 (12.74)	<0.001	0.918
Subepithelial	1.26 (0.87)	147.46 (23.70)	<0.001	0.89 (0.61)	208.93 (40.35)	<0.001	1.60 (1.60)	79.53 (17.12)	<0.001	0.036
IL-23										
Epithelial	2.68 (1.12)	189.08 (28.14)	<0.001	3.11 (2.12)	288.24 (46.57)	<0.001	2.30 (1.07)	79.47 (16.89)	<0.001	0.003
Subepithelial	1.21 (0.85)	250.12 (32.97)	<0.001	2.56 (1.74)	354.31 (53.82)	<0.001	0.00 (0.00)	134.97 (25.58)	<0.001	0.006

*Values are expressed as the number of positive cells, mean (standard error mean).
